# Sex differences in the association of skin advanced glycation endproducts with knee osteoarthritis progression

**DOI:** 10.1186/s13075-017-1226-z

**Published:** 2017-02-17

**Authors:** Charles B. Eaton, Maria Sayeed, Syeda Ameernaz, Mary B. Roberts, John D. Maynard, Jeffrey B. Driban, Timothy E. McAlindon

**Affiliations:** 1 Alpert Medical School of Brown University, Providence, USA; 2 School of Public Health of Brown University, Providence, USA; 3 Center of Primary Care and Prevention, Memorial Hospital of Rhode Island, 111 Brewster Street, Pawtucket, RI 02860 USA; 4 Department of Medicine, Memorial Hospital of Rhode Island, 111 Brewster Street, Pawtucket, RI USA; 5 Vera Light Inc., 800 Bradbury Dr SE # 217, Albuquerque, NM USA; 6 Division of Rheumatology, Tufts Medical Center, 800 Washington Street, Box 406, Boston, MA USA

**Keywords:** Advanced glycation endproducts, Knee osteoarthritis, Progression, Sex differences

## Abstract

**Background:**

The accumulation of advanced glycation endproducts in articular cartilage has been suggested as an etiologic factor in the development and progression of knee osteoarthritis (KOA).

**Methods:**

We conducted a prospective cohort study of skin advanced glycation endproducts (sAGEs) measured non-invasively by skin intrinsic fluorescence and the relationship between sAGE KOA progression in 160 men and 287 women in a sub-cohort of the Osteoarthritis Initiative at a single site. KOA progression was measured by yearly changes in Osteoarthritis Research Society International (OARSI)-defined joint space narrowing (JSN) and by yearly changes in joint space width (JSW) from baseline to 48 months. Sex-stratified repeated measures, mixed models to account for correlation between the knees within persons and adjusted for age, body mass index (BMI), Kellgren-Lawrence (KL) grade, beam angle and rim-to-rim distance were utilized.

**Results:**

Increasing tertiles of sAGE measured at 36 months were associated with greater JSN over 4 years in men but not in women. The percentage of knees with JSN at 48 months, by tertiles of sAGE, were 7.0%, 16.0% and 17.7% in men (*p* for linear trend = 0.03) and 11.4%, 14.4% and 8.4% in women (*p* for linear trend = 0.33). Using change in JSW as the outcome, a similar trend was found in men but it was not statistically significant in fully adjusted models and no association was found in women.

**Conclusion:**

This study provides preliminary evidence that sAGEs independent of age and BMI, are associated with knee JSN in men but not in women.

**Electronic supplementary material:**

The online version of this article (doi:10.1186/s13075-017-1226-z) contains supplementary material, which is available to authorized users.

## Background

Osteoarthritis (OA) is characterized by the progressive destruction of articular cartilage and is strongly and positively associated with chronological age, but the mechanism by which aging contributes to this increased susceptibility is largely unknown. Recently the hypothesis has been promulgated that accumulation of advanced glycation endproducts (AGEs) that are associated with cumulative glycemic exposure, oxidative stress and aging might explain some or most of this association [1]. Alterations in the extracellular matrix and loss of resident cells to repair this matrix damage induced by oxidative stress and AGEs have been suggested as explanations for the progressive articular damage associated with aging. AGEs are the endproducts of spontaneous reactions of reducing sugars with proteins, or non-enzymatic glycation found in tissues throughout the human body. These sugar-protein products are stabilized in humans through a group of complicated biochemical reactions (Maillard reactions) that produce a range of pigmented, fluorescent and glucose-derived protein cross-links that are collectively known as AGEs. Most AGEs have not been characterized in human systems; therefore a few markers of the non-enzymatic glycation process have been used to study their effects on human biology. AGEs form at accelerated rates in many tissues in patients with diabetes mellitus and therefore have been largely studied to better understand this disease and its complications [2–4]. AGEs have been associated with the several other age-related chronic diseases including Alzheimer’s dementia, and cardiovascular disease [5–8]. Pentosidine, a fluorescent AGE formed by lysine and arginine residues, is a highly sensitive marker of AGE and is often used to characterize the whole family of AGEs in human tissues. Pentosidine has been shown to accumulate in the collagen matrix of skin, lens, and articular cartilage [1].

Of particular importance in OA, low turnover tissues such as articular cartilage appear to be particularly sensitive to the accumulation of AGEs regardless of glucose metabolic status [1, 7]. Several lines of evidence have been reported that suggest that the accumulation of AGEs in articular cartilage might be an important mechanism in the association between aging and OA. First, articular chondrocytes express the receptors of advanced glycation endproducts in humans [9]. Second, in dog models of experimentally induced OA, elevated levels of AGEs lead to more severe OA based upon histologic grading, increased collagen damage and enhanced release of proteoglycans [10]. Third, human articular cartilage incubated in AGEs demonstrates a dose-dependent increase in AGEs that is associated with increased stiffness of the collagen network and decreases in the tensile strength of the collagen network suggesting that accumulation of AGE crosslinks might be an important putative mechanism in the development or progression of OA [11]. Senoult et al. [12] found increased levels of pentosidine, an advanced glycation endproduct in the serum and synovial fluid of patients with knee OA compared to controls. Both the serum and synovial fluid pentosidine levels correlated with synovial fluid cartilage oligomeric matrix protein (COMP) but not with radiographic staging of the knee OA. Vos et al. found in cross-sectional analysis that skin pentosidine was associated with increased severity and progression of knee and hip OA [13, 14].

This evidence adds additional support to the concept that AGEs may act as novel biomarkers of the incidence and progression of knee OA. However, not all studies are supportive of this hypothesis. Hunter et al. did not find correlation between urinary pentosidine and cartilage loss in the Boston Osteoarthritis of the Knee Study [15]. In addition, Vos et al. identified an inverse relationship of cartilage pentosidine levels and microscopic, histological and biochemical cartilage damage in samples obtained in total knee replacement [16].

Advanced glycation endproduct serum assays are not widely available due to a complex mixture of trace compounds in tissue proteins and the sophisticated and expensive techniques, such as gas chromatography and mass spectrometry, which are needed to reliably and precisely measure these products. However, when pentosidine is exposed to light in the near-UV and blue spectrum, it emits characteristic wavelengths of light called autofluorescence. Levels of the pentosidine found in skin biopsy specimens are highly correlated with collagen-linked fluorescence of the skin. Recently several groups have developed non-invasive means to measure skin fluorescence and thus indirectly measure skin AGE levels [17–19]. These skin fluorescence levels have been shown to be correlated with collagen-linked fluorescence of the skin and AGE levels in skin biopsy specimens in both human and animal models [20, 21].

We were therefore interested in exploring the association of skin fluorescence with the progression of knee OA by measuring changes in joint space narrowing (JSN) and changes in joint space width (JSW) over a 4-year period.

## Methods

### Cohort description

This is an approved ancillary study to the Osteoarthritis Initiative (OAI) performed at a single clinical center, the Brown University, Pawtucket, Rhode Island site (*n* = 1000 participants, 2000 knees). Detailed information about the OAI protocol is available (http://oai.epi-ucsf.org) [22]. Briefly, the OAI is a multi-center, longitudinal, prospective observational study of knee OA. The overall aim of the OAI is to develop a public domain research resource to facilitate the scientific evaluation of biomarkers for osteoarthritis as potential surrogate endpoints for disease onset and progression. In 2004–2006, the OAI collected baseline data from four study sites (i.e. Baltimore MD, Columbus OH, Pittsburgh PA and Pawtucket RI, USA) totaling 4796 patients with established OA or at risk of developing knee OA.

**Fig. 1:**
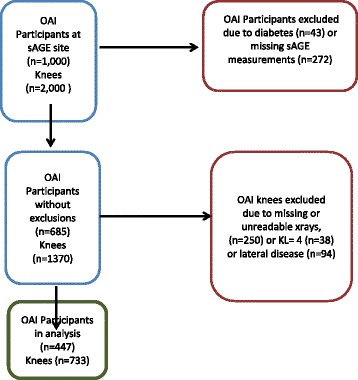
Flow diagram for determination of Ostearthritis Initiative (OAI) participants used in analysis. *sAGE* skin advanced glycation endproduct, *KL* Kellgren/Lawrence grade

Skin intrinsic fluorescent measurements were performed at 36 months at a single clinical center in Pawtucket RI in 728 of the 1000 participants. Knees were excluded from this analysis if they had (1) severe radiographic OA, as evidenced by a baseline Kellgren/Lawrence (K/L) grade of 4 (n = 38) or (2) primarily lateral JSN at any point from baseline to 48 months (n = 94). Additionally, specific follow-up knee data were excluded if there was missing or unsatisfactory knee positioning on the follow-up radiograph (n = 250). Unsatisfactory knee positioning was indicated if the difference in rim distance (from the tibial plateau to the tibial rim closest to the femoral condyle) between that follow-up visit and the baseline visit was >2 mm. This is done to minimize possible effects of knee position on measurement error in the measurement of joint space width (JSW). The analytic cohort comprised 447 participants (733 knees) with a K/L grade of 0–3, and who had skin AGE (sAGE) data available at 36 months (see flow diagram, Fig. 1). Repeated radiographic measurements at 12, 24, 36 and 48 months were included in this analysis. Due to potential alterations in sAGEs, 43 patients with diabetes mellitus were removed from the analytic dataset.

### Radiographic assessment of knees

In the OAI, current assessment of radiographs involved both semi-quantitative assessment of JSN and quantitative assessment of JSW. Using the semi-quantitative approach, JSN was scaled at grade intervals of 0.2 (i.e. 1.2, 1.4, etc.) and any change by grade 0.2 in the medial compartment was considered progression [23].

A quantitative approach was used to provide a precise measure of JSW in millimeters between the adjacent bones of the knee [24, 25]. Multiple JSWs were measured at fixed locations along the joint in the medial compartment, denoted as JSW(x), at intervals of 0.025 for x = 0.15–0.30. The reproducibility of this technique and the responsiveness to change have been documented elsewhere [26–28] including one study using OAI data, which demonstrated responsiveness that compared favorably to magnetic resonance imaging (MRI) [26]. We used medial JSW at x = 0.25 with the best responsiveness of change to quantify the progression of OA [28]. We defined the repeated measures of the changes in JSW from baseline to 12, 24, 36 and 48 months as one of the outcome variables. To account for changes in beam angle and alignment at each visit, which introduces measurement error in serial JSW measurement and JSN assessments, we also adjusted for changes of the beam angles and rim distances (from the tibial plateau to the tibial rim closest to the femoral condyle between follow-up visits and baseline). For these analyses, we used the publically available quantitative JSW measurements (version 06/17/2013, online at http://oai.epi-ucsf.org) and the semi-quantitative JSN readings (kXR_SQ_BU, version 06/17/2013, online at http://oai.epi-ucsf.org.

### Skin intrinsic fluorescence measurement

Levels of AGEs were determined using spectroscopic measurement of sAGEs at the 36-month visit [19, 21]. We measured sAGE using the SCOUT DS (VeraLight Inc., Albuquerque, NM). sAGE measures skin intrinsic fluorescence using a specifically designed fiber-optic probe that sends near-UV and blue excitation light to the forearm skin of the subject and the resulting skin fluorescence and diffuse reflectance is detected by a charge-coupled device array. In addition, a white-light-emitting diode (LED) illuminated the skin to measure the subject’s skin tone and correct the measured fluorescence for skin tone to yield skin intrinsic fluorescence.

The sequentially illuminated, excitation LEDs in the instrument have peak wavelengths of 375, 405, 420, 435, and 460 nm. The optical radiation emitted from the skin is dispersed in a modified research-grade spectrometer. A mathematical algorithm is applied to spectrum results to adjust for hemoglobin, skin pigmentation and light scattering. Thus, the subject-specific distortion of the emitted fluorescence is normalized by the measured skin tone to yield the intrinsic fluorescence. For our analysis, we used the excitation wavelength of 375 nm and emission wavelengths of 435–660 nm, which are correlated with crosslinks of collagen, flavin adenine dinucleotide (FAD) and nicotinamide adenine dinucleotide hydride (NADH) and thus, represent sAGEs and oxidative stress most likely associated with accumulation in articular cartilage. The output is scaled 0–100, with higher levels associated with higher levels of AGEs.

### Covariates

Sociodemographic factors measured at baseline included race/ethnicity, age, sex, smoking status, education years, and income. Race/ethnicity (African American, white or other race) was self-reported. Age, sex and smoking status (never, past or current) were self-reported. Body mass index (BMI) was measured by physical exam and was calculated from measured height and weight (weight (kg)/height (m)^2^) was classified into normal weight (BMI 18.5–24.9), overweight (BMI 25.0–29.9), or obese (BMI ≥30). Waist circumference was measured at the umbilicus twice and the average taken. sAGE levels were examined by smoking status and found to be equivalent in current and past smokers. The smoking status variable was restructured for further analysis into never smoked versus current and past smoking.

### Statistical analysis

The focus of the analysis was to assess the association between sAGEs and change in JSN and JSW over the study period. As sAGEs were not normally distributed we rank-ordered the data and categorized sAGEs into sex-specific tertiles. The outcomes were repeated measures of the change in JSN and JSW from baseline to 12, 24, 36 and 48 months. We tested for interaction by sex using the *F* test and found it was significant (*p* = 0.007 for JSN and *p* = 0.008 for JSW). Therefore, separate models of repeated measures for men and women were used to test the independent association between sAGEs and the change in JSN and JSW over time. Because of the hierarchical structure of the data (participant, knee, measures over time), linear mixed models were used to account for within-subject correlation and for the correlation of repeated measures at the knee for JSW and logistic mixed models for JSN. We additionally adjusted for beam angle and rim-to-rim distance to account for changes in JSW and JSN due to changes in position.

We evaluated crude models and models adjusted for age, BMI, and baseline K/L grade to evaluate the effect of increasing tertiles of sAGEs on JSN and JSW over the 4-year period from baseline to year 4. While smoking status and hypertension were associated with sAGE levels, they were not associated with progression of knee OA in men, and when added to our models in women they did not change the results of the analysis, and therefore these variables were not incorporated into our multiple variable models. All data analyses were conducted using SAS, version 9.2 (Cary, NC, USA).

## Results

sAGE scores ranged from 18.7 to 69.5 in men and 17.8 to 59.7 in women. We evaluated 160 men (255 knees) and 287 women (478 knees). Sex-specific ranges for each tertile of sAGEs and the cross-sectional associations between sAGEs and baseline characteristics are given in Table 1. Only older age and hypertension were associated with increasing levels of sAGEs in both sexes. Past and current smoking was associated with increasing levels of sAGEs in men only. Of note in women, the lowest tertile of sAGEs was associated with the greatest abdominal circumference and the percentage of women with obesity, contrary to the direction that was expected.

**Table 1:** Tertile of sAGEs and baseline characteristics by sex

	Men				Women				*P* value for sex
	Tertile 1	Tertile 2	Tertile 3		Tertile 1	Tertile 2	Tertile 3		
	18.7–28.0	28.1–33.7	33.9–69.5	*P* value	17.8–24.8	24.9 − 28.9	29.0–59.7	*P* value	
Subjects, *n*	56	50	54		104	86	97		
Knees, *n*	88	81	86		171	146	161		
BMI, mean (sd)	29.0 (3.4)	29.6 (4.1)	29.5 (3.8)	0.65	30.1 (5.4)	29.0 (5.4)	29.0 (5.5)	0.26	0.98
Age, years, mean (sd)	55.6 (7.7)	61.7 (8.5)	66.2 (8.8)	<0.01	58.8 (9.3)	63.1 (8.4)	64.6 (8.0)	<0.01	0.28
Ethnicity-Hispanic, *n* (%)	1 (1.8)	2 (4.0)	1 (1.9)	0.71	0 (0.0)	2 (2.3)	1 (1.0)	0.29	0.26
Race, *n* (%)									
White	54 (96.4)	49 (98.0)	48 (88.9)	0.21	100 (96.2)	85 (98.8)	91 (93.8)	0.01	0.45
Black	0 (0.0)	0 (0.0)	3 (5.6)		0 (0.0)	0 (0.0)	3 (3.1)		
Other	2 (3.6)	1 (2.0)	3 (5.5)		4 (3.8)	1 (1.2)	3 (3.1)		
Past or current smoking, *n* (%)	21 (37.5)	27 (54.0)	35 (64.8)	0.02	50 (48.1)	43 (50.0)	56 (57.7)	0.36	0.99
Abdominal circumference, mean (sd)	102.26 (9.83)	104.97 (11.28)	105.18 (10.99)	0.28	108.56 (13.53)	106.13 (14.02)	105.79 (12.78)	0.28	0.02
WC > sex cut, *n* (%)	30 (53.6)	33 (66.0)	37 (68.5)	0.22	100 (96.2)	79 (91.9)	91 (93.8)	0.45	<0. 01
Systolic BP, mean (sd)	122 (14.1)	125 (15.9)	128 (13.1)	0.13	122 (14.7)	123 (14.2)	124 (17.8)	0.70	0.12
Diastolic BP, mean (sd)	76 (9.1)	74 (10.4)	73 (9.5)	0.20	71 (8.1)	70 (8.7)	70 (10.6)	0.41	<0.01
HTN, *n* (%)	25 (44.6)	31 (62.0)	43 (79.6)	<0.01	48 (46.2)	53 (61.6)	58 (59.8)	0.06	0.18
Dyslipidemia, *n* (%)	14 (25.0)	16 (32.0)	21 (38.9)	0.29	25 (24.0)	24 (27.9)	32 (33.0)	0.37	0.42
BMI category, *n* (%)									
Normal	3 (8.9)	7 (14.0)	6 (11.1)	0.63	23 (22.1)	23 (26.7)	25 (25.8)	0.65	<0.01
Overweight	32 (57.1)	21 (42.0)	26 (48.2)		28 (26.9)	27 (31.4)	32 (33.0)		
Obese	19 (33.9)	22 (44.0)	22 (40.7)		53 (61.0)	36 (41.9)	40 (41.2)		
Knee K/L grade									
0–1	49 (55.7)	35 (43.2)	40 (46.5)	0.35	73 (42.7)	51 (34.9)	67 (41.6)	0.41	0.02
2–3	39 (44.3)	46 (56.8)	46 (53.5)		98 (57.3)	95 (65.1)	94 (58.4)		
Baseline joint space width, mean (se)	6.069 (0.164)	6.247 (0.170)	6.403 (0.166)	0.36	5.159 (0.114)	5.298 (0.116)	5.329 (0.108)	0.52	<0.01
Baseline joint space narrowing (*n*, %)									
OARSI 0	50 (56.8)	34 (42.0)	35 (40.7)	0.06	99 (57.9)	71 (48.6)	76 (47.2)	0.24	0.90
OARSI 1	23 (26.1)	27 (33.3)	28 (32.6)		38 (22.2)	44 (30.1)	48 (29.8)		
OARSI 2	15 (17.1)	20 (24.7)	23 (26.7)		34 (19.9)	31 (21.2)	37 (23.0)		

*BMI* body mass index, *BP* blood pressure, *OARSI* Osteoarthritis Research Society International, *HTN* Hypertension, *WC* Waist Circumference

Due to multiple exclusions and measurement of sAGEs at a single site, we were concerned about potential selection bias and issues of generalizability. We therefore compared our sample to those excluded and to the overall OAI sample (Additional file 1: Table S1). The analytic sample differed from those excluded at the site where sAGEs were measured in terms being a slightly younger age compared to the excluded sample, and we noted fewer changes in JSW over the 4 years of measurement in the analytic sample when compared to the excluded sample at the site of measurement of sAGEs. The participants at the site where sAGEs were measured differed from the eligible participants in the overall OAI sample in that a higher percentage were women, there were fewer African Americans, more past or present smokers, greater abdominal circumference, lower diastolic blood pressure, less hypertension, lower K/L grades and less change in JSW.

The percentage of men and women with worsening JSN are given by tertile of sAGEs in Table 2, comparing a crude model and fully adjusted models of age, BMI, K/L grade, rim distance and beam angle. In all models there was a monotonically increased relationship between worsening JSN and sAGEs in men but not in women, with 9%, 16.9% and 20.9% progression in men in the base models and 13.7%, 19.6% and 13.4% in women in the base models. Adjusting for age, BMI, and K/L grade individually in men had little effect on the estimates and with full adjustment the test for trend remained statistically significant (*p* = 0.03). For women the null results (*p* for trend = 0.93) persisted with adjustment for potential confounding (*p* for trend = 0.33).

**Table 2:** Change in joint space narrowing over 4 years by tertile of skin advanced glycation end products (sAGEs)

sAGEs	Crude model	Trend *p* value	Fully adjusted model	Trend *p* trend
Men				
Tertile 1: 18.7–28.0	9.02%	0.010	6.99%	0.028
Tertile 2: 28.1–33.7	16.88%		15.98%	
Tertile 3: 33.9–69.5	20.92%		17.72%	
Women				
Tertile 1: 17.8–24.8	13.67%	0.932	11.39%	0.328
Tertile 2: 24.9–28.9	19.6%		14.39%	
Tertile 3: 29.0–59.7	13.39%		8.44%

**Table 3:** Change in joint space width over 4 years by tertile of skin advanced glycation end products (sAGEs)

sAGEs	Crude model	Trend *p* value	Fully adjusted model	Trend *p* value
Men				
Tertile 1: 18.7–28.0	0.208 (0.058)	0.023	0.247 (0.060)	0.154
Tertile 2: 28.1–33.7	0.288 (0.059)		0.292 (0.058)	
Tertile 3: 33.9–69.5	0.384 (0.057)		0.374 (0.059)	
Women				
Tertile 1: 17.8–24.8	0.216 (0.034)	0.523	0.209 (0.035)	0.838
Tertile 2: 24.9–28.9	0.246 (0.036)		0.219 (0.036)	
Tertile 3: 29.0–59.7	0.184 (0.035)		0.194 (0.035)	

Mean change in joint space width (SE) in mm

Measured change in JSW at a fixed point in the medial tibia as a measure of progression of OA by tertile of sAGEs are given in Table 3, comparing crude, and fully adjusted models of age, BMI, and K/L grade. In the crude model in men there was a significant monotonically increased relationship between JSW by tertile of sAGEs (*p* for trend = 0.02). However in the full model (*p* for linear trend = 0.15) the relationship was no longer statistically significant. Similar to the JSN analysis, there was no relationship between tertiles of sAGEs and loss of JSW in women over serial yearly measurements.

We performed several sensitivity analyses, adding patients with diabetes mellitus to our study population, and only evaluating the white participants due to concerns that the skin autofluorescence would be affected by skin pigmentation. Including patients with diabetes mellitus did not materially affect our results, with a significant *p* value for trend for association between tertiles of sAGEs and JSN in men (*p* trend = 0.02) and no relationship in women (*p* = 0.27). Similarly, in comparing tertiles of sAGEs and changes in JSW there was a non-significant trend (*p* = 0.14) in men and no relationship in women (*p* = 0.84). Evaluating white participants only reduced the sample size, and while a similar result was found in terms of trends in men compared to women, they were no longer statistically significant (*p* = 0.08 and 0.39, respectively, for JSN and *p* = 0.26 and 0.94, respectively, for changes in JSW.

## Discussion

In this study of progression of knee OA, using semi-quantitative measurements (of JSN) we found that higher levels of skin AGEs were associated with progression of medial-tibial knee OA in men but not in women, and identified a trend using serial measurements of loss of JSW. These results provide preliminary evidence supporting the hypothesis that AGEs may play an important role in the progression of knee OA, particularly in men.

In the CHECK cohort, Vos et al. [13, 14] demonstrated an association between skin pentosidine and more severe knee OA at baseline, but only a modest association with progression of a combined score of hip and knee OA. Using skin biopsies and measurements of skin pentosidine levels, they did show a weak association (*r* = 0.167, *p* = 0.02) between skin pentosidine levels and K/L score for progression in the hip and knee. This relationship persisted when adjusted for age and BMI. They did not present any sex-specific findings or evaluate a sex-based interaction effect. Using measurements of urinary pentosidine, Hunter et al. did not find any association with cartilage loss in the knee [15]. However, the ability of urinary pentosidine in the setting of normal renal function to reflect the effect of AGEs on cartilage is unknown.

In our study, we identified progression of OA with increased skin AGE levels in men and not in women, suggesting sex differences in the effect of skin AGEs on the progression of OA. This finding could be spurious or could suggest that there are unique sex-specific mechanistic pathways that may lie behind such differences. Sex differences in the prevalence, incidence and severity of radiographic and clinical OA are well-described [29, 30], but specific examination of sex differences in MRI biomarkers or in biomarkers of joint metabolism are few, and results vary considerably [31, 32].

Men compared women have less prevalent and incident knee OA and less severe OA after the age of 55 years [29, 30]. MRI suggests that women have a smaller volume of lower knee cartilage compared to men [31]. Women have higher levels of bone resorption biomarkers [32] whereas men have higher levels of serum hyaluronan (HA), an indicator of synovial inflammation [33]. Karvonen-Guteirrez, Sowers and Heeringa [34] reported relationships between cardiometabolic risk factors and knee OA that were unique to each gender and to obesity status. In non-obese men, BMI and homeostasis model assessment-insulin resistance (HOMA-IR) and leptin, were the risk factors related to knee OA. In obese men, BMI was not related to knee OA, but HOMA-IR was strongly related to it. In non-obese and obese women, BMI continued to be a strong predictor of knee OA; however, HOMA-IR was inversely related to knee OA in obese women. In non-obese and obese men, leptin was found to be inversely related to the presence of radiographic OA, whereas leptin was positively associated with radiographic knee OA in obese women. These findings suggest that the effects of metabolic factors on knee OA are different in men and women, similar to our findings related to sAGEs. Indeed, insulin resistance, elevated insulin-like growth factor (IGF)-1, and AGEs are all related to body fat distribution and adipocyte biology, which might partially explain the gender difference found in our study [35–39].

### Strengths and limitations

The strengths of this study include the novel use of skin intrinsic fluorescence as a surrogate measure of sAGEs and the use of both JSN and JSW as measures of cartilage loss and thus, progression of knee OA. The limitations include the single measurement of skin intrinsic fluorescence at 36 months, while progression was determined yearly from time zero to 48 months, and thus, a clear temporal sequence potentially reflecting a cause and effect relationship is not present in this study. It is thought that skin fluorescence captures the cumulative exposure of AGEs in collagen and is relatively stable over time compared to serum or urinary pentosidine [17–20]. However, dietary AGEs, trace metals, cigarette smoking and other enzymatic co-factors may affect sAGEs and thus, the stability of a single measurement of sAGEs needs further study. It should, however, be noted that single measures of skin fluorescence are associated with increased risk of diabetes mellitus, cardiovascular disease, renal disease and mortality [2–6].

In addition, given the large number of exclusions and the study being performed at a single site, concerns about selection bias and generalizability are of concern. The lack of difference between included and excluded individuals and between knees for most covariates within the site of measurement of sAGEs is reassuring as it suggests that selection bias is unlikely to explain our results. The lesser changes in JSW in our analytic cohort compared to those excluded at the site of measurement of sAGEs would make it harder to find a significant association, so we may be underestimating the effect of sAGEs on changes in JSW.

We identified a stronger association using semi-quantitative measurement of JSN as a measure of progression compared to the quantitative measurement of changes in JSW, due to greater sensitivity to change using JSN. This may be related to the fact that JSN measurements use serial evaluation of knee radiographs by experienced musculoskeletal radiologists ordered by time, whereas JSW is measured independent of time using a computerized grid system. When comparing changes in JSW, subtle progression of OA that may be missed due to alignment issues obscuring these changes over the same time period may be picked up by experienced musculoskeletal radiologists.

The differences in sex, race and baseline K/L grade in our sample when compared to the overall OAI sample limits the generalizability of our findings and highlights the need for replication of our findings in additional cohorts. Indeed, while the sex differences appear strong they may be spurious due to the relatively small sample size. Elevated sAGEs are associated with pre-diabetes mellitus and diabetes mellitus [2–5]. To account for confounding related to the association between diabetes mellitus with OA and with sAGEs, we excluded participants with known diabetes mellitus; however, this reduced our sample size. We therefore performed additional sensitivity analyses including the 43 patients known to have diabetes mellitus, and the results were nearly identical.

## Conclusion

While biomechanical factors are clearly involved in the development and progression of knee osteoarthritis, systemic pathways including innate immunity, inflammation and advanced glycation endproducts are increasingly being recognized as playing a potentially important role in the natural history of osteoarthritis. This study adds credence to this view of the pathophysiology of knee osteoarthritis and suggests that future studies should be performed to examine the advanced glycation endproduct hypothesis.

## Additional file

Additional file 1: Table S1. Comparison of included/excluded but otherwise eligible* groups in OAI. *otherwise eligible means the OAI participant met all inclusion criteria (no diabetes, baseline and at least one followup xray, KL grade <4, no lateral knee disease) but didn't have a sAGE reading performed at 36 months. (DOC 66 kb)

## Abbreviations

AGEs: Advanced glycation endproducts
BMI: Body mass index
FAD: Flavin adenine dinucleotide
JSN: Joint space narrowing
JSW: Joint space width
K/L: Kellgren/Lawrence
KOA: Knee osteoarthritis
LED: Light-emitting diode
MRI: magnetic resonance imaging
NADH: Nicotinamide adenine dinucleotide hydride
OA: Osteoarthritis
OAI: Osteoarthritis Initiative
OARSI: Osteoarthritis Research Society International
sAGE: Skin advanced glycation endproducts
