# Supplementary material for: Sex differences in the association of skin advanced glycation endproducts with knee osteoarthritis progression
Source: Arthritis Res Ther. 2017 Feb 17;19:36. doi: 10.1186/s13075-017-1226-z (PMC5316210; doi:10.1186/s13075-017-1226-z)
Supplement: Additional file 1: Table S1. — Comparison of included/excluded but otherwise eligible* groups in OAI. *otherwise eligible means the OAI participant met all inclusion criteria (no diabetes, baseline and at least one followup xray, KL grade <4, no lateral knee disease) but didn't have a sAGE reading performed at 36 months. (DOC 66 kb) [file 13075_2017_1226_MOESM1_ESM.doc]

***Additional file 1: Table S1: Comparison of Included/Excluded but Otherwise Eligible* Groups in OAI***

|  | ***sAGE Sample*** | ***Otherwise Eligible* Sample: All Sites*** | ***p-value*** |  | ***Otherwise Eligible* Sample: Same Site*** | ***p-value*** |
| --- | --- | --- | --- | --- | --- | --- |
| *n* | *447* | *2179* |  |  | *117* |  |
| *# knees* | *733* | *3553* |  |  | *193* |  |
| *Person Level Data* |  |  |  |  |  |  |
| *Age (mean, sd)* | *61.7 (9.1)* | *61.4 (9.1)* | *0.4563* |  | *63.6 (10.1)* | ***0.0490*** |
| *BMI (mean, sd)* | *29.4 (4.9)* | *29.0 (4.8)* | *0.1038* |  | *29.7 (5.2)* | *0.4809* |
| *Gender (n, %)* |  |  | ***0.0067*** |  |  | *0.7164* |
| *Male* | *160 (35.8)* | *931 (42.7)* |  |  | *44 (37.6)* |  |
| *Female* | *287 (64.2)* | *1248 (57.3)* |  |  | *73 (62.4)* |  |
| *Hispanic (n, %)* | *7 (1.6)* | *23 (1.1)* | *0.3323* |  | *0 (0.0)* | *0.3544* |
| *Race (n, %)* |  |  | ***<0.0001*** |  |  | *0.7256* |
| *White* | *427 (95.5)* | *1689 (77.6)* |  |  | *109 (93.2)* |  |
| *Black* | *6 (1.3)* | *441 (20.3)* |  |  | *3 (2.6)* |  |
| *Other* | *14 (3.2)* | *49 (2.1)* |  |  | *5 (4.2)* |  |
| *Past or current smoking (n, %)* | *232 (51.9)* | *958 (44.0)* | ***0.0021*** |  | *62 (53.0)* | *0.8336* |
| *Abdominal circumference (mean, sd)* | *105.89 (12.60)* | *103.16 (12.79)* | ***<0.0001*** |  | *107.34 (13.33)* | *0.2744* |
| *WC > sex-specific cut point (n, %)* | *370 (82.8)* | *1592 (76.3)* | ***0.0029*** |  | *97 (82.9)* | *0.9731* |
| *Systolic BP (mean, sd)* | *124 (15.3)* | *125 (16.4)* | *0.0723* |  | *123 (13.3)* | *0.6899* |
| *Diastolic BP (mean, sd)* | *72 (9.5)* | *77 (10.2)* | ***<0.0001*** |  | *72 (9.2)* | *0.8322* |
| *HTN (n, %)* | *258 (57.7)* | *1370 (62.9)* | ***0.0408*** |  | *71 (60.7)* | *0.5624* |
| *Dyslipidemia (n, %)* | *132 (29.5)* | *538 (24.7)* | ***0.0325*** |  | *27 (23.1)* | *0.1672* |
| *BMI Category (n, %)* |  |  | *0.2709* |  |  | *0.7380* |
| *Normal* | *89 (19.9)* | *443 (20.4)* |  |  | *20 (17.1)* |  |
| *Overweight* | *166 (37.1)* | *884 (40.6)* |  |  | *47 (40.2)* |  |
| *Obese* | *192 (43.0)* | *849 (39.0)* |  |  | *50 (42.7)* |  |
| *Knee Level Data (one observation per knee)* | |  |  |  |  |  |
| *Knee KL Grade (n, %)* |  |  | ***0.0181*** |  |  | *0.1214* |
| *Grade 0-1* | *315 (43.0)* | *1330 (37.4)* |  |  | *69 (35.8)* |  |
| *Grade 2-3* | *418 (57.0)* | *2223 (62.6)* |  |  | *124 (64.2)* |  |
| *Knee Level Data (multiple observations per knee)* | |  |  |  |  |  |
| *JSW change (mean, se)* | *0.243 (0.019)* | *0.300 (0.009)* | ***0.0068*** |  | *0.347 (0.038)* | ***0.0131*** |
| *JSN progression (%)* | *16.14* | *13.91* | *0.0861* |  | *18.69* | *0.3074* |
